# Supplementary material for: Silk derived formulations for accelerated wound healing in diabetic mice
Source: PeerJ. 2021 Jan 8;9:e10232. doi: 10.7717/peerj.10232 (PMC7798629; doi:10.7717/peerj.10232)
Supplement: Supplemental Information 2 [file peerj-09-10232-s002.pdf]

A.

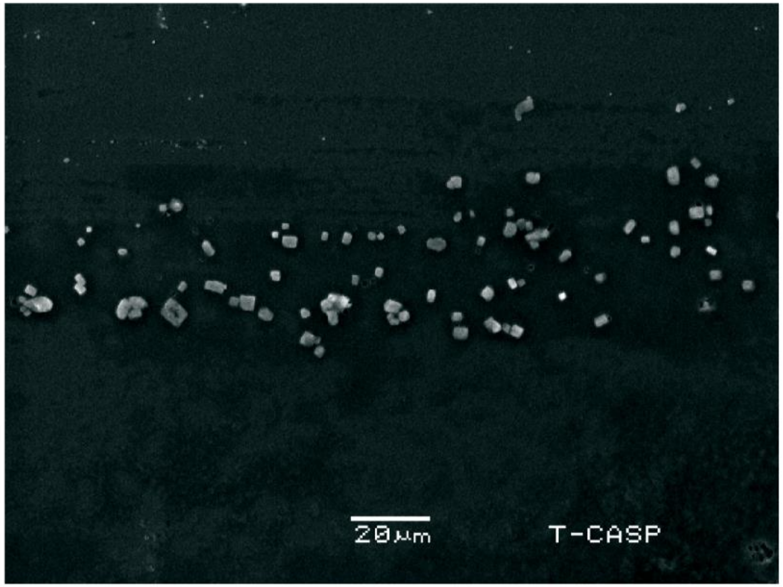

B.

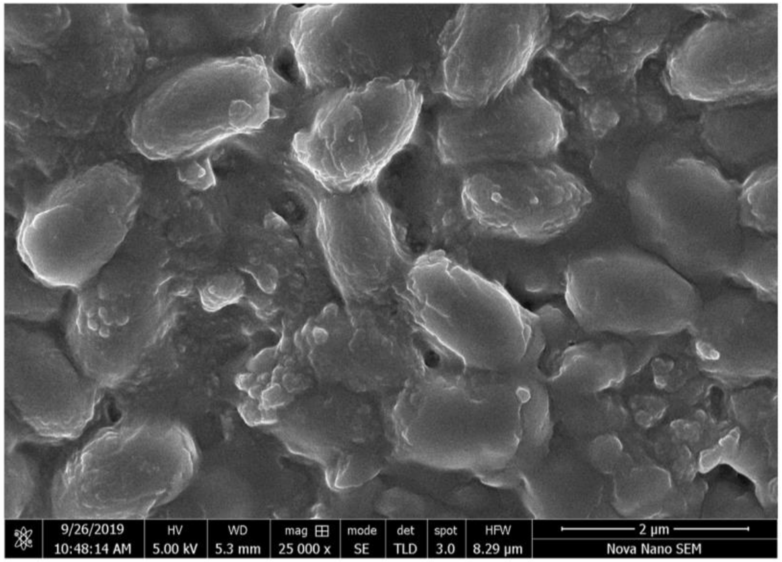

Supplemental Figure 01. Electron micrographs of Silk fibroin & Silk sericin. A. Electron micrograph of sonicated silk fibroin. **B.** Silk sericin micrograph.
